# Dysregulation of iron transport-related biomarkers in blood leukocytes is associated with poor prognosis of early trauma

**DOI:** 10.1016/j.heliyon.2024.e27000

**Published:** 2024-02-28

**Authors:** Zhusheng Feng, Yingnan Fan, Xiaofei Shi, Xu Luo, Jiangang Xie, Shanshou Liu, Chujun Duan, Qianmei Wang, Yuqin Ye, Wen Yin

**Affiliations:** a Department of Emergency, Xijing Hospital, The Air Force Medical University, Xi'an, China; b Department of Neurosurgery, Xijing Hospital, The Air Force Medical University, Xi'an, China; c Department of Neurosurgery, PLA 921th Hospital (Second Affiliated Hospital of Hunan Normal University), Changsha, China

**Keywords:** Trauma, Iron transport, Prognosis, Leukocyte, Gene expression

## Abstract

**Objective:**

The early targeted and effective diagnosis and treatment of severe trauma are crucial for patients' outcomes. Blood leukocytes act as significant effectors during the initial inflammation and activation of innate immunity in trauma. This study aims to identify hub genes related to patients' prognosis in blood leukocytes at the early stages of trauma.

**Methods:**

The expression profiles of Gene Expression Omnibus (GEO) Series (GSE) 36809 and GSE11375 were downloaded from the GEO database. R software, GraphPad Prism 9.3.1 software, STRING database, and Cytoscape software were used to process the data and identify hub genes in blood leukocytes of early trauma.

**Results:**

Gene Ontology (GO) analysis showed that the differentially expressed genes (DEGs) of blood leukocytes at the early stages of trauma (0–4 h, 4–8 h, and 8–12 h) were mainly involved in neutrophil activation and neutrophil degranulation, neutrophil activation involved in immune response, neutrophil mediated immunity, lymphocyte differentiation, and cell killing. Kyoto Encyclopedia of Genes and Genomes (KEGG) analysis showed that the DEGs were mainly involved in Osteoclast differentiation and Hematopoietic cell lineage. Sixty-six down-regulated DEGs and 148 up-regulated DEGs were identified and 37 hub genes were confirmed by Molecular Complex Detection (MCODE) of Cytoscape. Among the hub genes, Lipocalin 2 (LCN2), Lactotransferrin (LTF), Olfactomedin 4 (OLFM4), Resistin (RETN), and Transcobalamin 1 (TCN1) were related to prognosis and connected with iron transport closely. LCN2 and LTF were involved in iron transport and had a moderate predictive value for the poor prognosis of trauma patients, and the AUC of LCN2 and LTF was 0.7777 and 0.7843, respectively.

**Conclusion:**

As iron transport-related hub genes in blood leukocytes, LCN2 and LTF can be used for prognostic prediction of early trauma.

## 1 Introduction

Trauma is a major health issue worldwide that causes high morbidity and mortality. The most common causes of trauma include road injuries, falls, self-harm, exposure to natural forces, *etc* [1]. The early targeted and effective diagnosis and treatment of severe trauma are crucial for improving the patients' outcomes. For many years, physicians and researchers have discovered many methods to detect and manage high-risk trauma patients with poor prognosis [[2], [3], [4], [5]]. Although the currently utilized anatomic or physiologic scores can predict clinical prognosis, a trauma scoring system based on new biomarkers can provide useful clinical information in a more brand vision [6,7]. The initial physiological and pathophysiological processes of trauma are quite complicated and still unclear. Post-traumatic innate immune response disorders are usually considered indicators of poor outcomes [8]. Blood leukocytes that act as significant effectors during the initial inflammation and activation of innate immunity can maintain and regulate an appropriate inflammatory response at the earliest stage of trauma [9]. 'Inflammation and the host response to injury (Trauma)' as a large-scale collaborative research program reveals the host response involved in physiological and pathophysiological processes of severe trauma, from which we can acquire the existing microarray data of blood leukocytes in trauma patients.

With the aid of genomic and proteomic technologies, the function of hub biomarkers can be discovered and the molecular mechanisms involved in trauma can be probed [10,11]. Utilizing these new techniques, we can realize the early physiological and pathological changes that are vital for trauma prognosis at the DNA, RNA, and protein levels and find new biomarkers that would complement traditional trauma scores for trauma patients' prognostic prediction. Up to now, clinically applicable biomarkers in trauma are still insufficient. Therefore, seeking novel and available molecular biomarkers to guide effective diagnostic and therapeutic strategies for trauma patients is still urgent.

The objective of this study was to identify hub genes of blood leukocytes involved in the progression of trauma at the early stages and related to the trauma prognosis.

## 2 Materials and methods

### 2.1 Patients and samples

Blood samples were gathered from severe blunt trauma patients and healthy people who consented to blood sampling, and the eligible ages for the study were 16 years and older. The total cellular RNA of blood leukocytes was isolated, extracted, and hybridized to create a gene expression for each participant [12]. The inclusion criteria were as follows: (1) blunt trauma without isolated head injury; (2) absence of traumatic brain injury, defined as either AIS head <4 OR GCS motor >3 within 24 h of injury; (3) emergency department arrival ≤6 h from the time of injury; (4) base deficit ≥6 OR systolic blood pressure <90 mmHg within 60 min of emergency department arrival; (5) fully or partially intact cervical spinal cord. The primary outcome measures included the time to death, the change in gene expression after trauma injury, and the number and types of complications. Detailed information can be obtained in the 'Inflammation and the Host Response to Injury (Trauma)' research program at https://clinicaltrials.gov/ct2/show/NCT00257231.

### 2.2 Profiling of gene expression

The transcription profile datasets of circulating leukocytes in trauma were obtained from the National Center of Biotechnology Information (NCBI) Gene Expression Omnibus (GEO) database (https://www.ncbi.nlm.nih.gov/geo/). The accession number was GEO Series (GSE) 36809 (https://www.ncbi.nlm.nih.gov/geo/query/acc.cgi?acc=GSE36809) and GSE11375 (https://www.ncbi.nlm.nih.gov/geo/query/acc.cgi?acc=GSE11375). The datasets for GSE36809 and GSE11375 were based on GPL570 Platforms (HG-U133_Plus_2, Affymetrix Human Genome U133 Plus 2.0 Array). Both GSE36809 and GSE11375 originated from the studies of the 'Inflammation and the Host Response to Injury (Trauma)' research program [9.13], and included the whole blood leukocytes samples that were taken within 12 h after trauma. We can acquire the sampling time in GSE36809 and 28-day all-cause mortality in GSE11375.

Eighteen iron transport-related genes were obtained from the UniProt annotated keywords KW-0410 (https://www.uniprot.org/keywords/KW-0410). The gene list is as follows: FXN, HEPH, HFE, LCN2, LTF, MFI2, SCARA5, SLC11A1, SLC11A2, SLC22A17, SLC25A28, SLC25A37, SLC40A1, STEAP1, STEAP2, STEAP3, STEAP4, and TF.

### 2.3 Data preprocess and statistical analyses

R software (version 4.0.3) (https://cran.r-project.org/) and Bioconductor Packages (http://www.bioconductor.org/) (“GEOquery”, “hgu133plus2.db”, “limma”, “reshape2”, “ggplot2”, “ggfortify”, “clusterProfiler”, “pheatmap”, “org.Hs.eg.db”, “dplyr”, “AnnotationHub”, and “DO.db”) were used to process the downloaded files, and to convert, calibrate, standardize and log_2_ transform the data and reject the unqualified data [13]. Detailed descriptions and versions of all packages were listed in the Supplementary Table 1.

**Table 1:** Trauma 0–12 h Go terms enrichment analysis.

Ontology	ID	Description	Gene count	*p*-value
Trauma 0–4 h				
BP	GO:0042119	neutrophil activation	66	9.964218e-39
BP	GO:0043312	neutrophil degranulation	65	1.966599e-38
BP	GO:0002283	neutrophil activation involved in immune response	65	2.894459e-38
BP	GO:0002446	neutrophil mediated immunity	65	1.165772e-37
BP	GO:0030098	lymphocyte differentiation	26	9.317521e-10
BP	GO:0001906	cell killing	18	1.121058e-09
BP	GO:0032496	response to lipopolysaccharide	24	5.408086e-09
BP	GO:0072593	reactive oxygen species metabolic process	22	7.592859e-09
BP	GO:0002237	response to molecule of bacterial origin	24	1.148150e-08
BP	GO:0045088	regulation of innate immune response	27	3.865543e-08
Trauma 4–8 h				
BP	GO:0042119	neutrophil activation	65	1.486020e-34
BP	GO:0043312	neutrophil degranulation	63	2.341321e-33
BP	GO:0002283	neutrophil activation involved in immune response	63	3.374178e-33
BP	GO:0002446	neutrophil mediated immunity	63	1.259062e-32
BP	GO:0030098	lymphocyte differentiation	33	1.476740e-13
BP	GO:0042110	T cell activation	36	2.927899e-12
BP	GO:0001906	cell killing	21	1.981736e-11
BP	GO:0046631	alpha-beta T cell activation	19	3.312352e-11
BP	GO:0050727	regulation of inflammatory response	34	1.887580e-10
BP	GO:0001819	positive regulation of cytokine production	33	2.443026e-10
Trauma 8–12 h				
BP	GO:0042119	neutrophil activation	61	5.410134e-38
BP	GO:0043312	neutrophil degranulation	60	1.321963e-37
BP	GO:0002283	neutrophil activation involved in immune response	60	1.891841e-37
BP	GO:0002446	neutrophil mediated immunity	60	6.889076e-37
BP	GO:0042110	T cell activation	32	7.292497e-13
BP	GO:0030098	lymphocyte differentiation	28	7.585968e-13
BP	GO:0045088	regulation of innate immune response	31	1.975975e-12
BP	GO:0046631	alpha-beta T cell activation	18	2.917738e-12
BP	GO:0001906	cell killing	19	9.409966e-12
BP	GO:0002367	cytokine production involved in immune response	15	3.427358e-11

GO: Gene Ontology; BP: biological process.

### 2.4 Differentially expressed genes (DEGs) screenings

The gene identity corresponding to the probe name of the annotation package of R software was converted into a gene symbol and saved in a CSV file. We applied the R package “limma” to identify the DEGs between control and trauma during different times (0–4 h, 4–8 h, and 8–12 h) of the first 12 h in GSE36809 against the following criteria: (1) |log (Fold Change (FC))|＞2; (2) *p*-value＜0.05; and (3) false discovery rate (FDR) < 0.05 and to identify the DEGs between trauma survivors and trauma non-survivors in GSE11375 against the following criteria: (1) |logFC|＞1; (2) *p*-value＜0.05.

### 2.5 Gene Ontology (GO) and Kyoto Encyclopedia of Genes and Genomes (KEGG) pathway enrichment analysis

We conducted GO enrichment analysis and KEGG pathway analysis using the cluster profile to reveal the most significant pathways associated with the DEGs in trauma (criteria: *p*-value＜0.05, significantly enriched). The *p*-adjust was obtained by Benjamini and Hochberg (BH) FDR algorithm.

### 2.6 The protein-protein interaction (PPI) network construction

We used the STRING 11.0 database (http://string-db.org/) to analyze and visualize the interactive relationships among DEGs and download the PPI network [14]. Cytoscape (version 3.8.0) (https://cytoscape.org/) was used to construct the PPI network, and Molecular Complex Detection (MCODE) was used to calculate the prime module in the whole network [15]. We set the criteria as follows: Degree Cutoff≥2, Node Score Cutoff≥0.2, K-Core≥2, Max. Depth＝100. We used the Venn diagram to analyze the common genes (http://jvenn.toulouse.inra.fr/app/example.html) [16].

### 2.7 Statistical analysis

Data were presented as means ± standard deviation (SD) or median (interquartile range (IQR)). Kolmogorov-Smirnov test was used to evaluate the normal distribution of the data. The statistical data were processed using R software, GraphPad Prism 9.3.1 software (GraphPad Software, Inc). The area under the curve (AUC) of the receiver operating characteristic (ROC) curve was used to determine the predictive value of the hub genes, the predictive value of AUC is classified as low (AUC≤0.7), medium (0.7＜AUC≤0.9) and high (AUC＞0.9). The Pearson correlation coefficients were computed to measure the relationship between the hub genes, and the correlation (R) is classified as low (0.3＜R ≤ 0.5), medium (R 0.5＜R ≤ 0.8), and high (R＞0.8).

## 3 Results

### 3.1 DEGs in blood leukocytes of early trauma

**Fig. 1:**
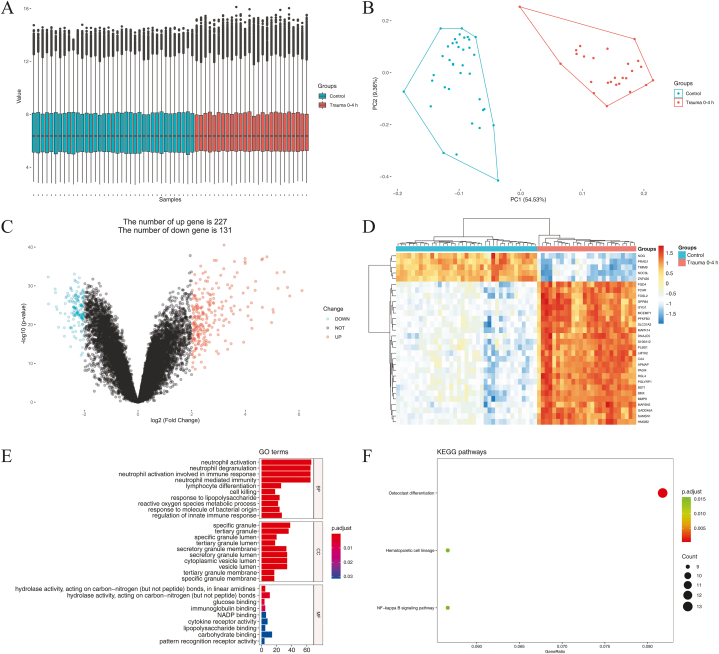
DEGs during 0–4 h in GSE 36809. (A) The standardization of the data. (B) PCA plot of the data. (C) Volcano plot of the DEGs. (D) Heatmap of the top30 DEGs. (E) Scatter plot of top 10 enriched GO terms of BP, CC, and MF, separately. (F) Scatter plot of the enriched KEGG pathways. DEGs: differentially expressed genes; GSE: Gene Expression Omnibus (GEO) Series; PCA: principal components analysis; GO: Gene Ontology; BP: biological process; CC: cellular component; MF: molecular function; KEGG: Kyoto Encyclopedia of Genes and Genomes.

**Fig. 2:**
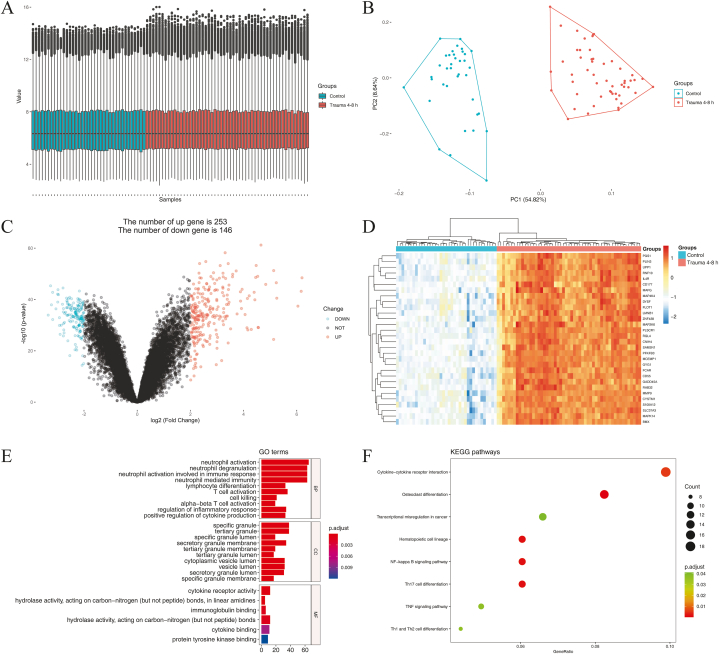
DEGs during 4–8 h in GSE36809. (A) The standardization of the data. (B) PCA plot of the data. (C) Volcano plot of the DEGs. (D) Heatmap of the top30 DEGs. (E) Scatter plot of top 10 enriched GO terms of BP, CC, and MF, separately. (F) Scatter plot of the enriched KEGG pathways.

**Fig. 3:**
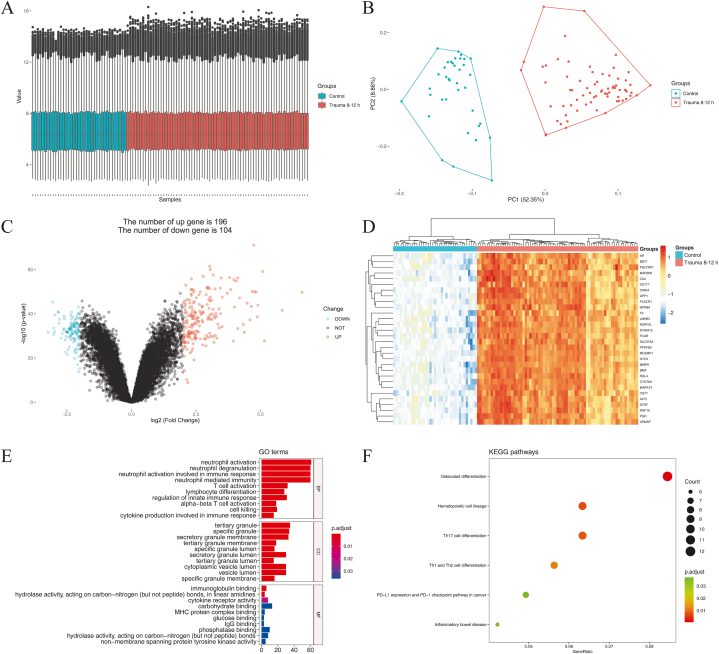
DEGs during 8–12 h in GSE36809. (A) The standardization of the data. (B) PCA plot of the data. (C) Volcano plot of the DEGs. (D) Heatmap of the top30 DEGs. (E) Scatter plot of top 10 enriched GO terms of BP, CC, and MF, separately. (F) Scatter plot of the enriched KEGG pathways.

One hundred and eighty-seven samples were analyzed during 0–12 h in GSE36809, including 37 samples in the control group and 26 (0–4 h), 53 (4–8 h), and 71 (8–12 h) samples in trauma groups. The characteristics of control and trauma patients during 0–12 h in GSE36809 were shown in Table S2, and there were no significant differences in gender and age among the groups (*p>*0.05). We used R software to standardize the sample data during 0–12h in GSE36809 and perform the principal components analysis (PCA) plot (Fig. 1A and B, Fig. 2A and B, and Fig. 3A and B). During 0–4 h, 358 genes displayed differential expression in blood leukocytes of early trauma, including 227 up-regulated genes and 131 down-regulated genes (|logFC|>2, *p<*0.05, and FDR <0.05) (Fig. 1C). During 4–8 h, 399 genes displayed differential expression, including 253 up-regulated genes and 146 down-regulated genes (|logFC|>2, *p<*0.05, and FDR <0.05) (Fig. 2C). During 8–12 h, 300 genes displayed differential expression, including 196 up-regulated genes and 104 down-regulated genes (|logFC|>2, *p* < 0.05, and FDR <0.05) (Fig. 3C). The top 30 DEGs cluster heatmaps in the early stages of trauma were shown in Figs. 1D–2D and 3D.

### 3.2 DEG enrichment analysis in blood leukocytes of early trauma

The TOP10 enriched GO terms and KEGG pathways in blood leukocytes of early trauma are shown in Fig. 1E and F, Fig. 2E and F, and Fig. 3E and F.

During 0–4 h, GO analysis results showed that the DEGs in blood leukocytes of trauma were mostly enriched in neutrophil activation, neutrophil degranulation, neutrophil activation involved in immune response, neutrophil mediated immunity, lymphocyte differentiation, cell killing, response to lipopolysaccharide, reactive oxygen species metabolic process, response to molecule of bacterial origin, and regulation of innate immune response (*p* < 0.05) (Table 1), and KEGG analysis results showed that the DEGs were mostly enriched in Osteoclast differentiation, Hematopoietic cell lineage, and NF-kappa B signaling pathway (*p* < 0.05) (Table 2).

**Table 2:** Trauma 0–12 h KEGG pathways enrichment analysis.

ID	Description	Gene count	*p*-value
Trauma 0–4 h			
hsa04380	Osteoclast differentiation	13	1.224162e-06
hsa04640	Hematopoietic cell lineage	9	1.350573e-04
hsa04064	NF-kappa B signaling pathway	9	1.971472e-04
Trauma 4–8 h			
hsa04380	Osteoclast differentiation	15	1.578245e-07
hsa04640	Hematopoietic cell lineage	11	1.265930e-05
hsa04064	NF-kappa B signaling pathway	11	2.031480e-05
hsa04659	Th17 cell differentiation	11	2.661395e-05
hsa04060	Cytokine-cytokine receptor interaction	18	1.107905e-04
hsa04668	TNF signaling pathway	9	9.197457e-04
hsa04658	Th1 and Th2 cell differentiation	8	1.057272e-03
Trauma 8–12 h			
hsa04380	Osteoclast differentiation	12	2.271418e-06
hsa04640	Hematopoietic cell lineage	9	5.633219e-05
hsa04659	Th17 cell differentiation	9	1.035980e-04
hsa04658	Th1 and Th2 cell differentiation	8	2.010953e-04
hsa05235	PD-L1 expression and PD-1 checkpoint pathway in cancer	7	9.283706e-04
hsa05321	Inflammatory bowel disease	6	9.362301e-04

KEGG: Kyoto Encyclopedia of Genes and Genomes.

During 4–8 h, GO analysis results showed that the DEGs were mostly enriched in neutrophil activation, neutrophil degranulation, neutrophil activation involved in immune response, neutrophil mediated immunity, lymphocyte differentiation, T cell activation, cell killing, alpha-beta T cell activation, regulation of inflammatory response, and positive regulation of cytokine production (*p* < 0.05) (Table 1), and KEGG analysis results showed that the DEGs were mostly enriched in Osteoclast differentiation, Hematopoietic cell lineage, NF-kappa B signaling pathway, Th17 cell differentiation, Cytokine-cytokine receptor interaction, TNF signaling pathway, Th1 and Th2 cell differentiation, and Transcriptional misregulation in cancer (*p* < 0.05) (Table 2).

During 8–12 h, GO analysis results showed that the DEGs were mostly enriched in neutrophil activation, neutrophil degranulation, neutrophil activation involved in immune response, neutrophil mediated immunity, T cell activation, lymphocyte differentiation, regulation of innate immune response, alpha-beta T cell activation, cell killing, and cytokine production involved in immune response (*p* < 0.05) (Table 1), and KEGG analysis results showed that the DEGs were mostly enriched in Osteoclast differentiation, Hematopoietic cell lineage, Th17 cell differentiation, Th1 and Th2 cell differentiation, PD-L1 expression and PD-1 checkpoint pathway in cancer, and Inflammatory bowel disease (*p* < 0.05) (Table 2).

Overall, GO analysis showed that the common DEGs of the early stages were involved in neutrophil activation, neutrophil degranulation, neutrophil activation involved in immune response, neutrophil mediated immunity, lymphocyte differentiation, and cell killing. KEGG analysis showed that the common DEGs of the early stages were involved in Osteoclast differentiation and Hematopoietic cell lineage.

### 3.3 Identification of hub genes in blood leukocytes of early trauma

**Fig. 4:**
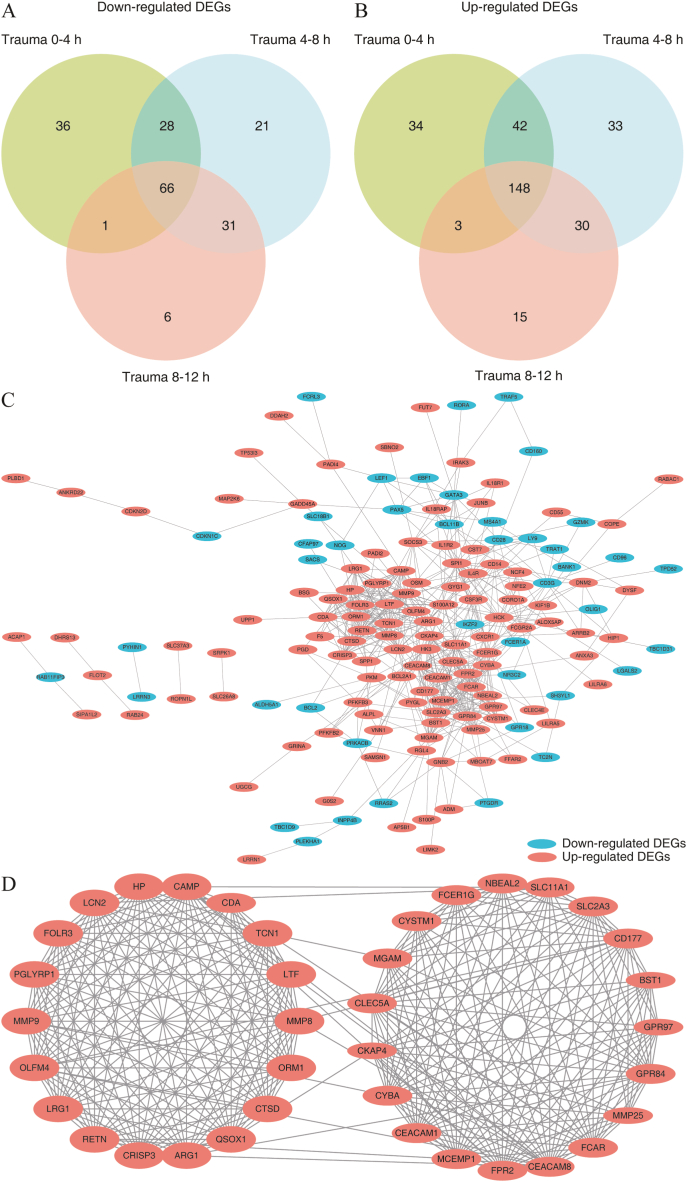
Identification of hub genes during 0–12 h in GSE36809. (A) Venn diagram of the down DEGs. (B) Venn diagram of the up DEGs. (C) PPI network of the common DEGs. (D) PPI network of the hub genes. PPI: protein-protein interaction.

We analyzed the gene list derived from DEGs of blood leukocytes during trauma 0–4 h, 4–8 h, and 8–12 h in a Venn diagram. We identified a total of 214 common genes in the early stages of trauma, including 66 down-regulated DEGs and 148 up-regulated DEGs (Fig. 4A and B). We used STRING and Cytoscape to analyze and visualize the interactive relationships among the common DEGs of different times (Fig. 4C). We analyzed the common DEGs by the MCODE of Cytoscape and identified 37 hub genes in the prime module, including NBEAL2, CYSTM1, SLC11A1, MCEMP1, CLEC5A, FCAR, GPR97, CD177, MMP25, CKAP4, CYBA, CEACAM1, SLC2A3, CDA, LCN2, CRISP3, FOLR3, QSOX1, MMP9, HP, LRG1, CAMP, ORM1, TCN1, CEACAM8, MMP8, CTSD, LTF, RETN, OLFM4, PGLYRP1, ARG1, FPR2, MGAM, BST1, FCER1G, GPR84 (Fig. 4D).

### 3.4 Identification of prognosis-related hub genes

**Fig. 5:**
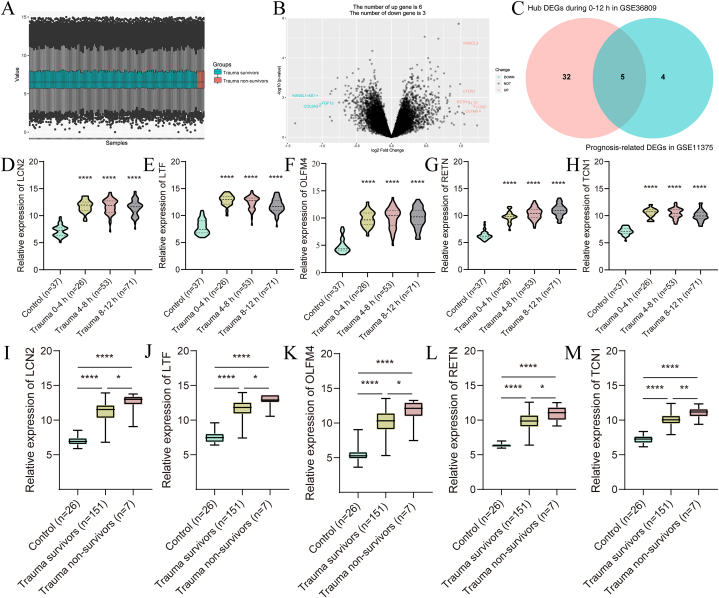
Identification of prognosis-related hub genes. (A) The standardization of the data of trauma survivors and non-survivors in GSE11375. (B) Volcano plot of the DEGs of trauma survivors and non-survivors in GSE11375. (C) Venn diagram of the prognosis-related hub genes. (D–M) Relative expression of the prognosis-related hub genes in control and trauma groups during 0–12 h in GSE36809. (J–N) Relative expression of the prognosis-related hub genes in control, trauma survivors, and trauma non-survivors group in GSE11375. *p < 0.05; **p < 0.01; ***p < 0.001; ****p < 0.0001.

We analyzed 158 samples between trauma survivors (n = 151) and trauma non-survivors (n = 7) in GSE11375. The characteristics of the control and trauma patients in the GSE11375 were shown in Table S3, and there were no significant differences in gender and age among the groups (*p* > 0.05). We standardized the samples of trauma in GSE11375 by R software (Fig. 5A). Between trauma survivors and non-survivors, nine genes displayed differential expression, including three down-regulated genes (COL9A3, FGF13, and KANSL1-AS1) and six up-regulated genes (LCN2, LTF, MUCL3, OLFM4, RETN, and TCN1) (|logFC|>1, *p* < 0.05) (Fig. 5B).

Five death-related DEGs were identified from the hub DEGs in trauma within 12 h by a Venn diagram, including LCN2, LTF, OLFM4, RETN, and TCN1 (Fig. 5C). In GSE36809, the five hub DEGs were significantly higher expressed in the trauma groups (0–4 h, 4–8 h, and 8–12 h) than the control group (*p* < 0.05) (Fig. 5D–H). In GSE11375, the five hub DEGs were also significantly higher in the trauma groups (survivors and non-survivors) than the control group and were higher in the trauma non-survivors group than the trauma survivors group (*p* < 0.05) (Fig. 5I-M).

### 3.5 Identification of iron transport-related hub genes

**Fig. 6:**
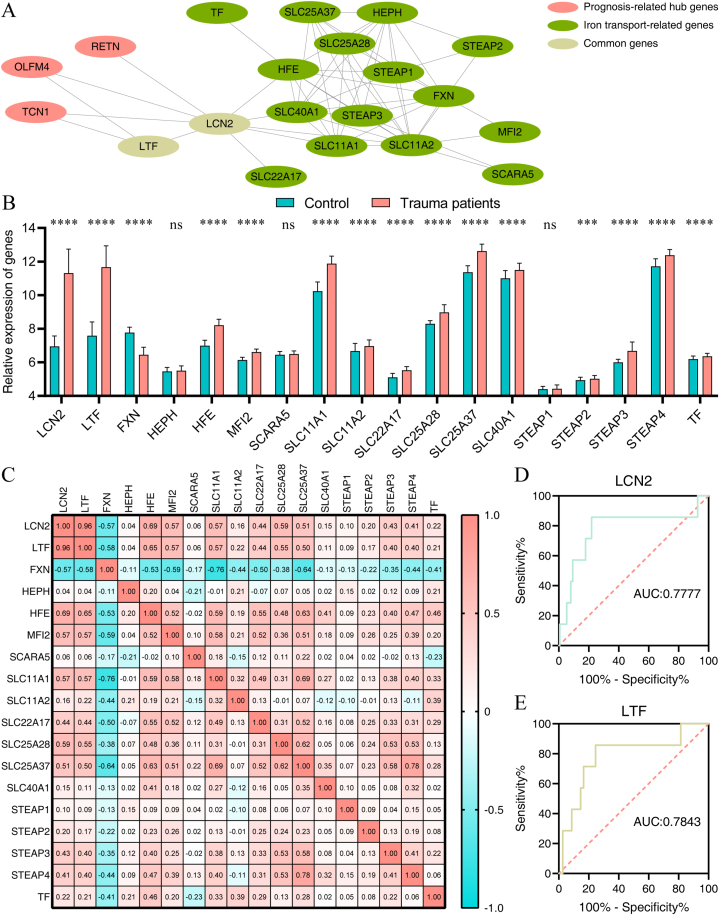
Identification of iron transport-related hub genes. (A) PPI network of the prognosis-related hub genes and the iron transport-related genes. (B) Relative expression of the iron transport-related genes in control and trauma groups in GSE11375. (C) Calculation of Pearson correlation coefficients to measure the relationship between the iron transport-related genes in GSE11375. (D–E) ROC analysis to evaluate the predictive value of the iron transport-related hub genes in trauma patients (survivors and non-survivors) of GSE11375. ROC: the receiver operating characteristic, AUC: area under the curve. ns: no significance; *p < 0.05; **p < 0.01; ***p < 0.001; ****p < 0.0001.

We used STRING to analyze the five prognosis-related hub genes, and the most closely connected annotated keyword in the UniProt was iron transport (kw-0410). We used Cytoscape to visualize the interactive relationships among the prognosis-related hub genes and the iron transport-related genes, and there were two common genes (LCN2 and LTF) in the PPI Network. (Fig. 6A). Fourteen iron transport-related genes (LCN2, LTF, HFE, MFI2, SLC11A1, SLC11A2, SLC22A17, SLC25A28, SLC25A37, SLC40A1, STEAP2, STEAP3, STEAP4, and TF.) were significantly higher and FXN was significantly lower in the trauma group than that in the control group (Fig. 6B). A high positive correlation was observed between LCN2 and LTF (R: 0.96). A moderate positive correlation was observed in LCN2 and LTF with HFE, MFI2, SLC11A1, and SLC25A28, and a moderate negative correlation was observed in LCN2 and LTF with FXN (0.5＜R ≤ 0.8) (Fig. 6C). We evaluated the predictive value of LCN2 and LTF for the poor prognosis of trauma patients in GSE11375 using the ROC analysis. It revealed that LCN2 and LTF had moderate predictive values (0.7＜AUC≤0.9) for the poor prognosis of trauma patients, and the AUC of LCN2 and LTF was 0.7777 and 0.7843, respectively (Fig. 6D and E).

## 4 Discussion

Identification of hub genes in blood leukocytes has been applied to liver diseases, infectious diseases, Parkinson's disease, cancer, and coronary artery diseases [[17], [18], [19], [20], [21]]. Previous studies have shown that the obvious alterations of the leukocyte transcriptome in trauma occurred from the initial 12 h to days and weeks. The genomic changes of blood leukocytes in trauma could induce the activation of pattern recognition and antigen presentation and provide useful clinical information for the prediction of prognosis [9,22]. Although it is known that the early changes of blood leukocytes are crucial for trauma patients' prognosis, the specific genes of blood leukocytes that participate in the early trauma and are related to trauma prognosis are still unclear.

At present, we can more thoroughly excavate hub genes through the bioinformatics technique and reveal the early physiological and pathological changes that are vital for trauma prognosis. In this study, we first analyzed the gene expression of blood leukocytes at the early stages of trauma (0–4 h, 4–8 h, and 8–12 h). The PCA plots, volcano plots, and cluster heatmaps of DEGs show apparent differences between the control and trauma groups. Significantly, there are more up-regulated DEGs than down-regulated DEGs in all early stages of trauma. Among the top 30 DEGs of the early stages, the down-regulated DEGs (NOG, PRAG1, TIMM9, NOC3L, and ZNF420) were only detected at the earliest stage of trauma (0–4 h). The GO analysis shows that the DEGs of all early stages are mainly involved in neutrophil activation, neutrophil degranulation, neutrophil activation involved in immune response, neutrophil mediated immunity, lymphocyte differentiation, and cell killing. The KEGG analysis shows that the DEGs of all early stages are mainly involved in Osteoclast differentiation and Hematopoietic cell lineage. According to our study, the gene expression levels of blood leukocytes at the early stages of trauma mostly reflect the RNA changes from neutrophils (PMN), future research can take single-cell sequencing technology to look for improved biomarkers of trauma outcome.

We identified 37 hub genes of blood leukocytes in the early stages of trauma (0–12 h), which concentrate on two groups and are all up-regulated DEGs (GSE36809). Nine DEGs (GSE11375) between trauma survivors and non-survivors were identified, and LCN2, LTF, OLFM4, RETN, and TCN1 are hub genes associated with poor prognosis in blood leukocytes of early trauma.

Many studies focused on the relationship between the blood concentration of the hub biomarkers and the prognosis of trauma. NGAL which was initially found in neutrophils is a 25-kD protein encoded by the LCN2 gene and belongs to the lipocalin family. It plays a vital role in innate immunity by sequestering iron-containing siderophores to limit bacterial growth [23,24]. The high blood concentration of LCN2/NGAL in trauma patients was considered to be a predictor of mortality [25,26], acute kidney injury (AKI) [[27], [28], [29]], the need for renal replacement therapy (RRT) [[28], [29], [30]], traumatic brain injury (TBI) severity [31], multiple organ dysfunction syndromes (MODS) [30] and renal failure [26]. RETN has an extremely stable and high-order multimeric structure and has a pleiotropic role in metabolism, inflammation, and diseases [32]. Elevated plasma RETN level was found on hospital admission and on days 1, 2, 3, 5, and 7 after TBI, and was considered to be associated with TBI mortality [33]. Lactotransferrin or lactoferrin (LTF) is a member of the transferrin family, and its protein product is found in the secondary granules of neutrophils. Its protein is a major iron-binding protein in milk and body secretions. Through antimicrobial activity, LTF plays a crucial part in the non-specific immune system [34,35]. Elevated serum LTF was associated with postoperative infectious complications in patients with acute traumatic spinal cord injury [36], and pretreatment with LTF reduced the incidence of trauma-hemorrhagic shock-induced gut injury [37]. Olfactomedin 4 (OLFM4) is a member of the olfactomedin family. Its encoded protein is an extracellular matrix glycoprotein and an antiapoptotic factor promoting tumour growth and cell adhesion [38,39]. Olfactomedin 4-positive neutrophils were upregulated and affected the inflammatory response after hemorrhagic shock [40]. Transcobalamins (TCS) are cobalamin carrier proteins and are elevated during trauma [41]. Transcobalamin 1 (TCN1) is expressed in various tissues and secretions, and its encoded protein is a significant constituent of secondary granules in neutrophils and can promote cobalamin transport into cells [42].

In our study, the five prognosis-related hub genes were connected with iron transport closely, and LCN2 and LTF were involved in iron transport. A high positive correlation was observed between LCN2 and LTF, and a moderate correlation was observed in LCN2 and LTF with other iron transport-related genes such as HFE, MFI2, SLC11A1, SLC25A28, and FXN. The ROC analysis showed that LCN2 (0.7777) and LTF (0.7843) had a moderate predictive value for the poor prognosis of trauma patients.

Iron is an essential trace element and is abundantly expressed in haemoglobin and myoglobin and the monocyte-macrophage system of many tissues. The normal operation of many fundamental physiological processes depends on iron transport [43,44]. Iron transport is associated with ferroptosis and the disturbances of iron transport play a vital role in traumatic injury [45,46]. As an iron metabolic gene, the interference of LCN2 could enhance ferroptosis and apply in cancer therapy [47]. LTF plays an important role in iron transport and can regulate the quantity of iron absorption [48].

The limitations still existed in this study. Even if the filter criteria were set to |logFC| > 1 and *p* < 0.05, only 9 DEGs were identified (GSE11375), the DEG changes between trauma survivors and non-survivors were not as obvious as the changes between the control and trauma groups. Besides, further studies with in-depth clinical verification of our findings are still needed. In future research, the combined application of the hub genes and traditional trauma scores for the prognosis would be beneficial to the clinical diagnosis and treatment of trauma patients, and the prediction efficiency of the hub genes for TBI and non-TBI is worth further vising. There is a close link between LCN2 and LTF, and the exact mechanism of the hub genes of iron transport in early trauma needs further validation. New sequencing technology such as single-cell RNA sequencing technology can be applied to explore the efficiency of the hub genes in different types of blood leukocytes for prognostic prediction in trauma patients.

## 5 Conclusion

As iron transport-related hub genes in blood leukocytes, LCN2 and LTF can be used for prognostic prediction of early trauma.

## Funding

This study was funded by the 10.13039/501100001809National Natural Science Foundation of China (81871587), the Key Scientific Research Project of Military Logistics (BWS21J002), the Natural Science Foundation of Hunan Province (2023JJ30432), the Excellent Youth Science Research Project of Hunan Education Department (22B0095) and the 10.13039/501100017596Natural Science Basic Research Program of Shaanxi Province (2022JM-437).

## Ethical approval

Detailed information can be obtained in the 'Inflammation and the Host Response to Injury (Trauma)' research program (https://clinicaltrials.gov/ct2/show/NCT00257231).

## Data availability

All data generated or analyzed during this study are included in this published article and its supplementary information files.

## Declaration of competing interest

The authors declare that they have no known competing financial interests or personal relationships that could have appeared to influence the work reported in this paper.
