# Supplementary material for: Dysregulation of iron transport-related biomarkers in blood leukocytes is associated with poor prognosis of early trauma
Source: Heliyon. 2024 Feb 28;10(5):e27000. doi: 10.1016/j.heliyon.2024.e27000 (PMC10923684; doi:10.1016/j.heliyon.2024.e27000)
Supplement: Multimedia component 1 [file mmc1.docx]

**TABLE S1:** The details of all packages used in the study.

| Name | Description | Version |
| --- | --- | --- |
| GEOquery | Get data from NCBI Gene Expression Omnibus (GEO) | 2.56.0 |
| hgu133plus2.db | Affymetrix Human Genome U133 Plus 2.0 Array annotation data (chip hgu133plus2) | 3.2.3 |
| limma | Linear Models for Microarray Data | 3.44.3 |
| reshape2 | Flexibly Reshape Data: A Reboot of the Reshape Package | 1.4.4 |
| ggplot2 | Create Elegant Data Visualisations Using the Grammar of Graphics | 3.3.1 |
| ggfortify | Data Visualization Tools for Statistical Analysis Results | 0.4.10 |
| clusterProfiler | statistical analysis and visualization of functional profiles for genes and gene clusters | 3.16.0 |
| pheatmap | Pretty Heatmaps | 1.0.12 |
| org.Hs.eg.db | Genome wide annotation for Human | 3.11.4 |
| dplyr | A 'dplyr' Back End for Databases | 1.4.4 |
| AnnotationHub | Client to access AnnotationHub resources | 2.20.0 |
| DO.db | A set of annotation maps describing the entire Disease Ontology | 2.9 |

| **TABLE S2:** Characteristics of control and trauma patients during 0-12 h in the GSE36809 | | | | | | | |
| --- | --- | --- | --- | --- | --- | --- | --- |
| **Parameter** | **Control (n=37)** | **Trauma 0-4 h (n=26)** | **Trauma 4-8 h (n=53)** | **Trauma 8-12 h (n=71)** | | ***p*-value** |  |
| Sex (male/female) | 20/17 | 17/9 | 28/25 | 49/22 | | 0.2249^a^ |  |
| Age (year) | 30.22±8.51 | 34.85±11.25 | 34.21±10.59 | 34.45±11.58 | | 0.2018^b^ |  |
| ^a^Data were analyzed by the Fisher' exact test; ^b^Data were analyzed by the one-way ANOVA. | | | | |  |  |  |

| **TABLE S3:** Characteristics of control and trauma patients in the GSE11375 | | | | |  |
| --- | --- | --- | --- | --- | --- |
| **Parameter** | **Control (n=26)** | **Trauma survivors (n=151)** | **Trauma non-survivors (n=7)** | ***p*-value** | |
| Sex (male/female) | 17/9 | 92/59 | 6/1 | 0.3943^a^ | |
| Age (year) | 29.88±10.20 | 33.68±11.25 | 33.00±9.90 | 0.2747^b^ | |
| ^a^Data were analyzed by the Fisher' exact test; ^b^Data were analyzed by the one-way ANOVA. | | | | |  |
